# BMT for Myelodysplastic Syndrome: When and Where and How

**DOI:** 10.3389/fonc.2021.771614

**Published:** 2022-01-06

**Authors:** Akriti G. Jain, Hany Elmariah

**Affiliations:** ^1^ Fellow, Hematology Oncology, H. Lee Moffitt Cancer and Research Institute, Tampa, FL, United States; ^2^ Department of Blood and Marrow Transplant and Cellular Immunotherapy, H. Lee Moffitt Cancer and Research Institute, Tampa, FL, United States

**Keywords:** myelodysplastic syndromes, revised international prognostic scoring system, allogeneic stem cell transplantation, therapy related MDS/AML, bone marrow failure

## Abstract

Myelodysplastic syndromes (MDS) are a diverse group of hematological malignancies distinguished by a combination of dysplasia in the bone marrow, cytopenias and the risk of leukemic transformation. The hallmark of MDS is bone marrow failure which occurs due to selective growth of somatically mutated clonal hematopoietic stem cells. Multiple prognostic models have been developed to help predict survival and leukemic transformation, including the international prognostic scoring system (IPSS), revised international prognostic scoring system (IPSS-R), WHO prognostic scoring system (WPSS) and MD Anderson prognostic scoring system (MDAPSS). This risk stratification informs management as low risk (LR)-MDS treatment focuses on improving quality of life and cytopenias, while the treatment of high risk (HR)-MDS focuses on delaying disease progression and improving survival. While therapies such as erythropoiesis stimulating agents (ESAs), erythroid maturation agents (EMAs), immunomodulatory imide drugs (IMIDs), and hypomethylating agents (HMAs) may provide benefit, allogeneic blood or marrow transplant (alloBMT) is the only treatment that can offer cure for MDS. However, this therapy is marred, historically, by high rates of toxicity and transplant related mortality (TRM). Because of this, alloBMT is considered in a minority of MDS patients. With modern techniques, alloBMT has become a suitable option even for patients of advanced age or with significant comorbidities, many of whom who would not have been considered for transplant in prior years. Hence, a formal transplant evaluation to weigh the complex balance of patient and disease related factors and determine the potential benefit of transplant should be considered early in the disease course for most MDS patients. Once alloBMT is recommended, timing is a crucial consideration since delaying transplant can lead to disease progression and development of other comorbidities that may preclude transplant. Despite the success of alloBMT, relapse remains a major barrier to success and novel approaches are necessary to mitigate this risk and improve long term cure rates. This review describes various factors that should be considered when choosing patients with MDS who should pursue transplant, approaches and timing of transplant, and future directions of the field.

## Introduction

Myelodysplastic syndromes (MDS) are a group of hematologic malignancies characterized by a combination of bone marrow (BM) dysplasia, cytopenias and risk of leukemic transformation (1). Other complications accompanying MDS include risk of infections, bleeding, iron overload, and cardio-pulmonary compromise (2). MDS is more prevalent in older adults with a median age at diagnosis of 77 years (2). Based on SEER (surveillance, epidemiology and end results program) data, the age-adjusted annual incidence of MDS in the United States was 5.6 per 100,000 in 2010, which declined to 4.0 per 100,000 in 2015. The incidence of MDS increases with advancing age. The long-term overall survival (OS) of patients with MDS is 31.3% at 5 years (2). However there is a large disparity in OS of MDS patients based on specific prognostic disease characteristics. Causes of death were evaluated in a large retrospective study showing that 47% of patients died due to acute myeloid leukemia (AML) progression, 27% due to infectious complications, 10% from bleeding, 16% due to reasons unrelated to their disease process (3).

In recent years, elucidation of the biology of MDS has led to approvals of new therapies such as hypomethylating agents that prolong survival but do not offer cure (4). Allogeneic blood or marrow transplantation (alloBMT) remains the only potential curative treatment and offers an OS benefit in eligible high risk MDS patients. However, alloBMT is associated with a risk of transplant related (TRM) due to infections, graft versus host disease (GVHD), chemotherapy related toxicities, rejection and relapse. With the introduction of reduced intensity conditioning regimens and increasing use of human leukocyte antigen (HLA) mismatched donors, alloBMT is being used more frequently for treatment of MDS (5). Still, the potential benefit of alloBMT may be negated in patients at high risk for TRM and relapse, so careful evaluation by a transplant specialist is necessary to identify suitable candidates. In this review we describe the indications and timing of transplant, transplant approaches, and post-transplant outcomes of patients with MDS.

## Clinical and Laboratory Features

### A. Cytopenias

The hallmark of MDS is bone marrow failure which occurs due to selective growth of somatically mutated clonal hematopoietic stem cells (6). Requirement of packed red blood cell (PRBC) or platelet transfusions are the major causes of morbidity in MDS. About 80-85% patients have anemia with a hemoglobin (Hb) of 8-10 g/dL (40%) and <8 g/dL (10%) at diagnosis (7). Approximately, 25% patients are transfusion dependent at diagnosis. Transfusion dependence is defined as requirement of 4 units of PRBCs or greater within a 8 week period by the International Working Group (8). Thrombocytopenia at diagnosis is seen in about 40% MDS patients and about 15% of patients die from bleeding without disease progression (7). Neutropenia is also seen in 20-25% of patients with MDS, leading to potential severe infections (7). 

### B. Cytogenetics

Cytogenetics in MDS are useful both for diagnosis and risk stratification of MDS patients. Most of the cytogenetic abnormalities that occur in MDS involve unbalanced changes leading to loss or gain of significant amount of chromosomal material such as deletion (del) 5q, monosomy 7, trisomy 8, and deletion 20q (9). The presence of specific MDS-associated cytogenetic abnormalities such as monosomy 7, del(7)q, del(5)q and i(17)q are sufficient to make a diagnosis of MDS in a patient with cytopenia even if dysplasia is not seen (1). Additionally, as validated by Schanz et al., the following cytogenetic changes can accurately risk stratify 91% of MDS patients: del(11q) and −Y are very good (median OS, 60.8 months); normal, del(5q), del(12p), del(20q) and double abnormalities including del(5q) are good (median OS, 48.6 months); del(7q), +8, i(17)(q10), +19, +21, any other single abnormality, independent clones, double abnormalities not harboring del(5q) or −7/del(7q) are intermediate (median OS, 26.0 months); inv(3)/t(3q)/del(3q), −7, double abnormalities including −7/del(7q), and complex (ie, three abnormalities) are poor (median OS, 15.8 months); complex (ie, > three abnormalities) are very poor (median OS, 5.9 months) (10).

### C. Somatic Mutations

The pathophysiology of MDS is associated with somatic mutations in multiple genes that are required for cell cycle regulation, RNA splicing, DNA transcription or methylation, or tumor suppression along with chromosomal aberrations (11). It is hypothesized that a driver mutation provides a hematopoietic stem cell (HSC) survival and proliferative advantage leading to clonal expansion and progression to MDS. A median of 3 somatic mutations are identified in each MDS patient (12–14). Some of these mutations are being utilized as actionable targets in trials to improve treatment options.

**Figure 1:**
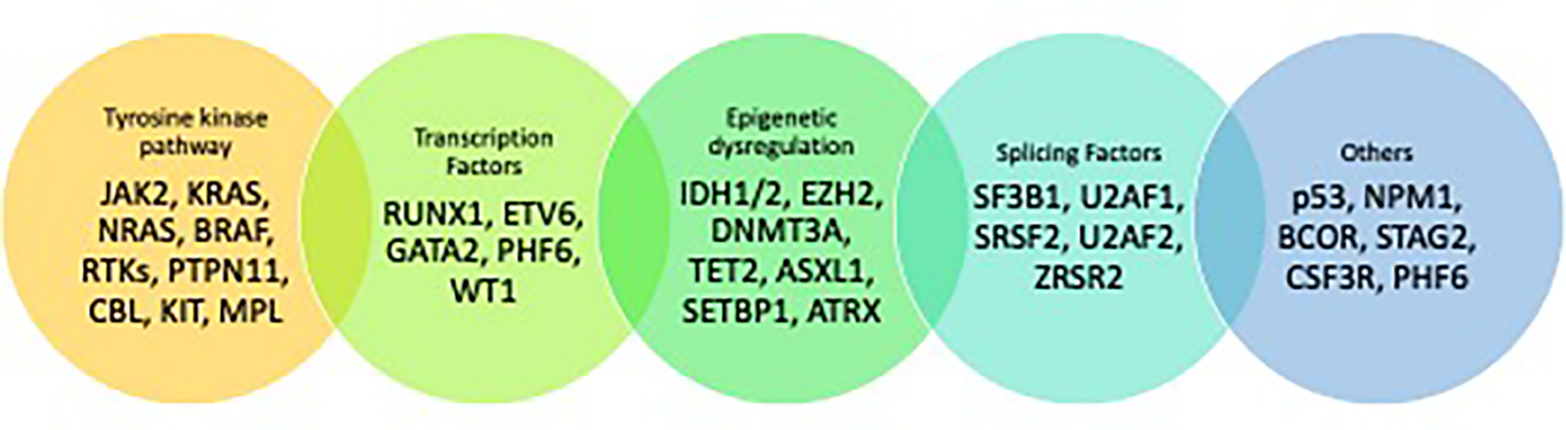
Mutational landscape of MDS (15).

Mutations in MDS can be classified based on their function (**Figure 1**) (12, 13, 16). The most common mutations seen in MDS include those involving spliceosome function such as *SF3B1* (24.5%), *SRSF2* (11.8%) and *U2AF1* (6.6%), mutations involving DNA methylation such as *TET2* (22.9%) and *DNMT3A* (10.3%), and histone modification such as *ASXL1* (12.9%). In patients with MDS, *SF3B1* mutations herald a good prognosis when associated with ringed sideroblasts and low blast counts. Conversely, *RUNX1, ASXL1*, and *TP53* have an adverse impact on prognosis (13, 17). In patients with “low risk” MDS (LR-MDS) with del(5q), up to 20% patients may harbor *TP53-*mutation (18). In these patients, the presence of a *TP53*-mutated clone at a variable allelic frequency (VAF) of 10% or more can confer a higher risk of progression and poor survival (19, 20).

### D. Prognostic Models

The prognosis of MDS patients is heterogenous in regards to expected time to AML transformation and OS. Determination of the prognosis for each patient is essential for guiding appropriate treatment. Multiple prognostic models have been developed including the International prognostic scoring system (IPSS), revised international prognostic scoring system (IPSS-R), WHO classification-based scoring system (WPSS) and MD Anderson prognostic scoring system (MDAPSS) (21, 22). The IPSS categorizes patients into four categories low, intermediate-1, intermediate-2 and high risk (23). In 2012, the IPSS was updated with more refined cytogenetic categories and divided patients with MDS into five sub groups and two broad categories: LR-MDS (≤3.5 points) and high risk (HR) MDS (>3.5 points) (24). The median OS varies significantly for the 5 risk groups: very low 8.8 years, low 5.3 years, intermediate 3 years, high 1.6 years and very high 0.8 years.

A critical deficiency of these predictive models is the absence of somatic mutations in the models; because of this, some patients deemed LR-MDS by the IPSS-R score may progress more rapidly than predicted. A recent paper by Nazha et al. aimed to create a personalized prediction model to help predict survival and leukemia transformation to help guide management in such patients (25). Somatic mutations in seven genes were prognostically significant, and mutations in multiple genes led to worse outcomes. The model outperformed the IPSS and IPSS-R scores for prognostic accuracy. Development of this model is a significant accomplishment in the field, and we anticipate widespread clinical application in the coming years.

Because the prognosis of MDS varies widely, anywhere from a few months to many years, alloBMT is not recommended for all patients with this disease. For HR-MDS patients, BMT is beneficial in light of their short expected survival. Because the IPSS and IPSS-R do not include somatic mutations, patients may be misclassified as having LR-MDS when, in fact, their disease will behave more aggressively due to high risk somatic mutations such as *RUNX1, ASXL1*, and *TP53*. Hence, the approach to treatment, including alloBMT, should follow a HR-MDS paradigm in such patients. The new personalized prediction model may offer better prognostication, but the role of this model for selecting candidates for alloBMT requires further validation.

## Role and Considerations for Allogeneic Transplant

### A. AlloBMT Indications

Risk stratification dictates the management of MDS. The goals of management for patients with LR-MDS are improving quality of life and cytopenias. For patients with HR-MDS, treatment focuses on delaying disease progression and improving survival. In this setting, the two treatment options that have shown survival benefit are hypomethylating agents (HMA) and alloBMT (4, 5). However, alloBMT is the only treatment that can offer cure for MDS and hence should be considered for all eligible patients with high-risk disease features.

**Figure 2:**
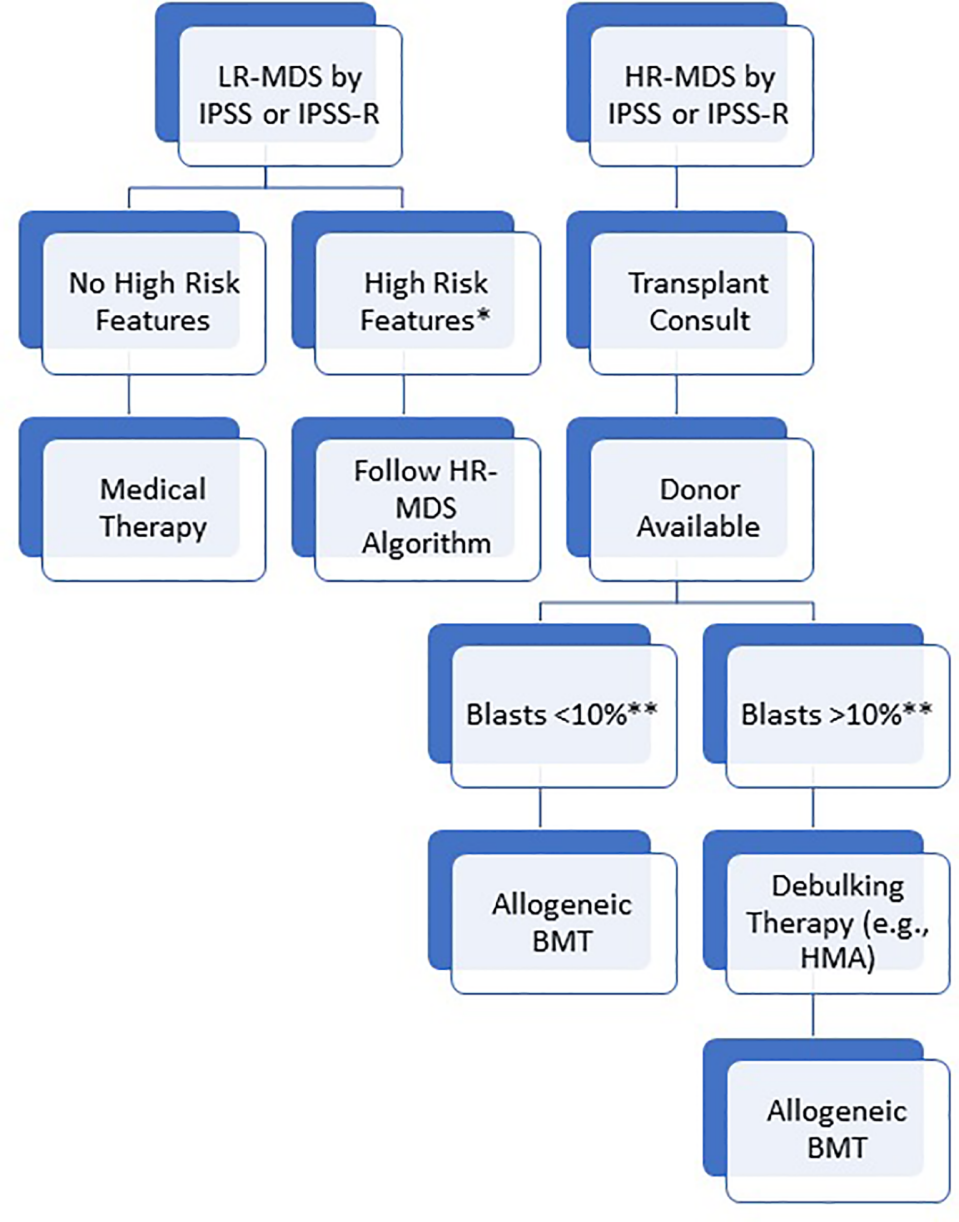
Suggested algorithm for allogeneic BMT. Patients are initially risk stratified based on IPSS or IPSS-R. For high risk patients, transplant consult should be pursued immediately and patients should proceed to transplant as soon as blasts are < 10%. For low risk patients, BMT should still be considered in the presence of high risk features (*high risk somatic mutations including TP53, ASXL1, and RUNX1; Blasts > 10%; transfusion dependence or severe neutropenia despite medical therapy; hypomethylating agent failure). **While a blast percentage below 10% is acceptable to proceed with transplant, the specific threshold is debated and institution dependent. Some centers may consider bridging medical therapy for patients with low blast percentage to maintain low disease burden during the donor search. For patients with an indication, allogeneic BMT should be pursued as soon as the blasts are adequately low/reduced.

Since it is difficult to randomize patients to alloBMT vs chemotherapy due to ethical reasons and donor availability, several biological assessment trials have compared alloBMT to HMA or best supportive care (26, 27). In HR-MDS patients of 50-70 years, Robin et al. compared alloBMT to no transplant based on availability of a matched donor and found 4-year OS was improved in the alloBMT group (37% versus 15%, p=0.02) (26). Similarly, the “VidazaAllo” study recently compared patients between 55-70 years of age who were treated with 4-6 cycles of azacitidine and then either underwent alloBMT or continued azacitidine (28). The 3-year event free survival (EFS) was better in the alloBMT cohort (34% vs 0%; p<0.0001), though OS was not significantly different in this trial (50% vs 32.5%; p=0.12). To address whether alloBMT provides benefit in older patients, Nakamura et al. recently studied 384 MDS patients up to 75 years of age assigned to BMT versus no BMT arms according to the availability of a matched donor within 90 days of study registration (27). They reported a significantly higher adjusted 3-year OS rate in the donor arm compared to the no-donor arm (47.9% vs 26.6%; p=0.0001). The 3-year leukemia free survival (LFS) was also higher in the donor arm (35.8% vs 20.6%; p=0.003). In summary, prospective studies have confirmed that alloBMT offers a survival benefit to patients with HR-MDS and a transplant consultation should be pursued for all such patients who are eligible for aggressive treatment, including fit elderly patients (**Figure 2**).

In patients with LR-MDS, life expectancy without transplant is on the order of years and, thus, the risks of alloBMT generally outweigh the potential benefits. However, some patients with LR-MDS based on IPSS or IPSS-R scoring should be considered for transplant in the setting of other high risk features including high risk clonal mutations such as *TP53*, in the presence of significant bone marrow fibrosis, intolerance or contraindication to available therapies, and transfusion dependent patients who fail to achieve a hematologic response even after best available therapy (29). Additionally, because treatment options are limited after HMA and subsequent outcome are poor, our practice is to offer alloBMT to any patient with HMA failure regardless of disease risk stratification (30). Median survival after HMA failure is <6 months, but outcomes are best for patients who are able to receive an alloBMT (30, 31). In a study on 277 patients post HMA failure alloBMT was pursued in 37 patients in whom median OS was 19 months (30). This was shown to be superior to other management strategies or supportive care. After treatment with intensive chemotherapy (ICT), the duration of remission and response status determine outcomes post alloBMT (32).

### B. AlloBMT Eligibility

While alloBMT is recommended in patients with HR-MDS, up to 90% of patients are presumed to be ineligible without a formal transplant evaluation due to age (33). Limiting to fit patients under 65 years, 97% undergo alloBMT (33). However, in recent years, increased accessibility to alternative donors and the advent of reduced intensity conditioning (RIC) have allowed more HR-MDS patients to be transplant eligible, regardless of age (34). Indeed, as recent data has prospectively confirmed an OS benefit with transplant even in patients up to 75 years, age should not be a primary consideration for transplant eligibility. Instead, a more individualized approach is needed with consideration of relapse risk, functional status, frailty, comorbidities, overall life expectancy, and goals of care rather than chronologic age (5).

A retrospective study using Center for International Blood and Bone Marrow Transplant Research (CIBMTR) data on 1080 patients who underwent RIC alloBMT showed that age did not significantly impact TRM, relapse, disease free survival (DFS) or OS if other factors including hematopoietic cell transplantation-specific comorbidity index (HCT-CI) were similar (35). Another similar study of 1333 MDS patients greater than 50 years old showed that age (>60 years vs. 50-60) did not significantly affect post alloBMT survival (RR 1.0, 95% CI 0.90-1.27). In this cohort, 62% of patients received RIC (36). In a study of 688 MDS patients greater than 65 years who were compared to 592 MDS patients between 55 and 64 years, Atallah et al. found that age did not affect OS (HR, 1.09; 95% CI, 0.94-1.27; P = 0.23) or TRM (HR, 1.19; 95% CI, 0.93-1.52; P = 0.16) after adjusting for excess risk for mortality in the older group. In addition, in the Bone Marrow Transplant Clinical Trials Network (BMT CTN) 1102 study, Nakamura et al. also showed that benefit of alloBMT was seen across all subgroups including patients above 65 years of age (27). Hence, with modern RIC regimens, alloBMT should be offered to all fit patients without a definitive upper age limit.

While fitness should be prioritized over chronological age for determining transplant eligibility, there is no “gold standard” metric to evaluate fitness for transplant. Assessment of the patient’s functional or performance status using Karnofsky or Eastern Cooperative Oncology Group (ECOG) scale is done prior to pursuing transplant as these assessment do correlate with OS after transplant. Frailty or assessment of the patient’s physical fitness or physiological age is another important factor that impacts outcomes after alloBMT. Frailty can be assessed using objective measures like the clinical frailty scale (CFS) (37). In a study on 118 MDS patients, Sakatoku et al. reported that CFS (≥ 5 vs. < 5; hazard ratio [HR], 3.37; P = .002) independently predicted OS on multivariate analysis (38). The HCT-CI score is used when evaluating comorbidities and a score of 3 to 4 and ≥ 5 have been shown to have significantly higher risk of mortality (39, 40). BMT CTN 1704 is an ongoing study combining pre-transplant comorbidity, geriatric assessment and other biomarkers such as C-reactive protein (CRP) and albumin to help predict TRM after alloBMT (41). In summary, physiologic age, frailty, and comorbidities, rather than chronologic age, should be primary considerations for determining alloBMT eligibility (42).

### C. Disease Related Prognostic Factors

While high risk features are indications for alloBMT in MDS, high risk features also impact the likelihood of cure after alloBMT. Accurate assessment of the post-transplant prognosis is of utility for patients to make informed decisions balancing the benefits versus risks of alloBMT. Della Porta et al. studied 519 patients who underwent alloBMT for MDS and showed that the high risk IPSS-R category and monosomal karyotype (MK) were independently correlated with relapse and inferior survival after alloBMT (43). The rates of 5-year OS noted in this study for patients with good, intermediate, poor and very poor cytogenetic risk were 48%, 37%, 28% and 15% respectively (p=0.003). The incidences of relapse for the same prognostic risk groups were 16%, 30%, 43%, and 41%, respectively (p = 0.001), while TRM was similar across the groups. MK independently predicted poor OS and high relapse rate. The 5-year OS of MK patients was only 10%, which was shown to be significantly worse than that of patients without MK (P <.001), whereas the 5-year cumulative incidence of relapse was 49%, which was reported to be significantly greater than that of patients without MK (P <.001) (43). Another study by Scheid et al. attempted to validate IPSS-R at the time of transplant rather than at diagnosis and found that the median OS from transplant was predictable according to IPSS-R: very low 23.6 months, low 55.0 months, intermediate 19.7 months, high 13.5 months, very high 7.8 months (P<0.001) (44).

Bone marrow fibrosis especially higher grade of fibrosis, myelofibrosis (MF) 3-4 is associated with inferior survival post alloBMT, higher rates of relapse post alloBMT and also delayed engraftment (45, 46). Kroger et al. showed that the cumulative incidence of engraftment at day +30 in patients without fibrosis was 93% and was significantly lower in patients with mild or moderate fibrosis (89%) and severe fibrosis (75%) (P=0.009) (46). This study also reported that patients with severe BM fibrosis had inferior survival irrespective of any other variables (HR 1.9; p=0.006) (46).

The burden of transfusions also affects outcome post alloBMT. It has been shown that transfusion dependency along with multilineage dysplasia is associated with increased TRM in MDS patients that undergo transplantation (HR 1.56; p=0.037) (47).

For alloBMT candidates, another risk stratification model that is widely utilized to predict OS is the disease risk index (DRI) (48). The DRI was validated for multiple hematologic malignancies, including MDS, in a study on 13,131 patients through CIBMTR who underwent transplant between 2008 and 2010 (49). For MDS, the DRI stratifies patients into one of four survival risk groups based on cytogenetics and disease status at the time of transplant. Despite successfully stratifying survival outcomes, the utility of the DRI is weakened by the absence of metrics related to TRM. In a recent study from Spain, Fernandez-Caballero studied a combination of risk scores (HCT-CI and DRI) to predict prognosis in 175 patients (50). They reported that a combination of DRI 0-1 and HCT-CI 0-2 showed a higher OS compared to DRI 0-1 and HCT greater than or equal to 3 (45% vs 36%; p 0.041) (50). The combination of these scores might better predict OS based though this needs to be validated in more patients.

As somatic mutations guide prognosis and high-risk mutations are considered an indication for transplant, many investigators have evaluated the predictive value of these mutations on post-BMT outcomes. In a study of 401 MDS/AML patients who underwent transplant, Della Porta et al. found that 87% of patients carried one or more oncogenic mutations. Mutations that impacted outcomes included *TP53* (HR for OS 2.54; p=0.004, HR for relapse 3.12; p=0.003), *ASXL1* (HR for OS 2.09; p=0.021, HR for relapse 2.41; p=0.029) and *RUNX1* (HR for OS 1.96; p=0.031, HR for relapse 2.46; p=0.038), irrespective of the IPSS-R (51). A subsequent larger study by Lindsley et al. included 1514 MDS patients identified in the CIBMTR. *TP53* was seen in 19% of patients and was associated with inferior survival (HR 1.71; p<0.0001) and earlier relapse (HR 2.03; p<0.001). The median variant allele frequency (VAF) for *TP53* mutation was 10% (range 2-86%) and a VAF of 10% or greater was associated with shorter survival (HR for death 1.28; p=0.07) (52). Additionally, mutations in the RAS pathway and *JAK2* were also associated with shorter survival. In another study on 87 MDS patients, mutations in *TP53* (HR, 2.30; P = .027), *TET2* (HR, 2.40; P = .033), or *DNMT3A* (HR, 2.08; P = .049) were reported to be associated with shorter OS after alloBMT (17).

Aside from prognostic significance, it remains to be determined how best to approach transplant modifications in the setting of specific mutations. In particular, because of the extremely high risk of relapse and low DFS associated with *TP53* mutations, some transplant centers have chosen to avoid transplant for this disease, arguing that the high risk of transplant related complications are not worthwhile in the context of a disease with such low potential for long term cure. However, most centers continue to offer transplant to these patients as the outcome without transplant is uniformly death related to MDS. Notably, the analysis by Lindsley et al. suggested that more intensive conditioning may result in worse outcomes due to a higher risk of TRM without a reduction in relapse, presumably due to *TP53* MDS being refractory to chemotherapy. Because of the low potential for cure, when available, transplant for *TP53* mutated MDS should ideally be pursued in the context of a clinical trial focused on relapse reduction (17, 29, 53).

### D. Donor Type

Donor availability and compatibility also play an important role in determining outcomes. In a recent study, Novak et al. reported 90% OS at 5 years for patients with low and intermediate-1 risk MDS with a good performance status and available matched sibling donor (MSD) or matched unrelated donor (MUD). They reported that the most important factor determining OS post alloBMT in their cohort of patients was HLA (human leukocyte antigen) matched versus mismatched donor (5 year OS 86% versus 55%; P = 0.008) (54). This finding has also been replicated in a transplantation specific risk model created by the European Group for Blood and Marrow Transplantation (EBMT) (55). Hence when available, HLA matched siblings and matched unrelated donors are preferred for patients with MDS undergoing alloBMT. After HLA matching, age, gender and CMV (cytomegalovirus) status are also considered when following standard donor selection criteria (5). In the matched donor setting, the source of hematopoietic stem cell sources has been studied and showed that peripheral blood stem cell (PBSC) grafts yield more rapid engraftment and higher risk of chronic graft-versus-host disease (GVHD), but no difference in OS or DFS (56–58). Given the similarity of long term survival outcomes, the choice of marrow versus PBSC is largely institution dependent with no clear standard approach.

Because patients with MDS are often elderly, their siblings are also typically elderly and at high risk for comorbidities that deem them unfit to be BMT donors. If MUDs are not available for such patients then alternative donor options may be considered, including HLA haploidentical relatives, umbilical cord blood transplants (UCBT), and mismatched unrelated donors. Grunwald et al. recently compared a total of 603 MDS patients in the CIBMTR database who underwent RIC MUD versus RIC haploidentical alloBMT between 2012 to 2017. They reported higher relapse (HR 1.56; p=0.0055), 2-year relapse rate (48% vs 33%) and lower DFS (29% vs 36%) with HLA haploidentical donors. However, there was no difference in OS (HR 0.94; p=0.65), 2 year OS (46% vs 44%). Rates of GVHD, both acute and chronic, were lower in patients who received HLA haploidentical donor alloBMT (59). UCBT is also an acceptable alternative for patients who do not have a matched sibling or unrelated donor. In a study on 176 MDS patients, a 3 year OS and RFS was 31% and 28% was reported. However the success of UCBT was hampered by the high TRM of about 40% at 3 years (60).

### E. Treatment for MDS Preceding AlloBMT

The optimal approach to treatment prior to alloBMT is not completely defined. For patients with increased blasts, treatment with either HMA or cytotoxic chemotherapy to reduce the blasts percentage is common practice. Evidence supporting this approach is based largely on a prospective study showing that increased blasts at transplant were associated with worse survival (26). However, this study design did not confirm whether reducing the blast percentage in patients with excess blasts results in outcomes similar to patients who never had excess blasts. Data supporting a reduction in blast percentage is otherwise limited to retrospective and single arm studies that suggest improved outcomes when the blast percentage is reduced to no more than 10% and ideally less than 5% (61–64). Achieving a reduction in blast percentage may be of particular importance for patients undergoing RIC prior to alloBMT as the lower intensity of chemotherapy is less likely to clear residual excess blasts (61). In patients without excess blasts, HMA may be used as a bridging therapy during the donor search. However, transplant should not be delayed to complete a pre-defined number of cycles of HMA as demonstrated in the VidazaAllo study where patients were treated with 4-6 cycles of HMA prior to transplant. In that study, 33% of patients who started HMA did not proceed to alloBMT due to disease progression, drug related adverse events, or new comorbidities (28). While specific strategies are debated, our approach is to attempt to debulk patients with excess blasts and proceed to transplant as soon as the bone marrow blasts are <10% (5). Notably, while blast percentage has been a key metric for transplant eligibility, future studies should also elucidate the prognostic significance of reducing or clearing the burden of somatic driver mutations prior to transplant.

Beyond blast reduction, some retrospective studies have evaluated the impact of the choice of pre-transplant treatment on post-transplant outcomes. A prior study using a Markov model to determine survival related to treatment approach and disease risk showed that in high risk disease, HMA use prior to transplant is associated with a 2-year gain of life expectancy, especially in older patients. They also showed that treatment with HMAs in intermediate risk disease was associated with superior OS than waiting until disease progression to high or very high risk disease (65). Various retrospective studies have compared HMA to intensive induction strategies and have reported similar outcomes (5, 66, 67). Gerds et al. compared azacitidine to induction chemotherapy (ICT) in a cohort of MDS and AML patients and found that OS, TRM and relapse free survival (RFS) were similar in both groups. Relapse rate was also similar in both groups after adjusting for disease risk (66). In another study, Damaj et al. showed that the rates of OS, EFS, relapse and TRM were similar for patients treated with azacitidine alone, induction chemotherapy alone or azacitidine preceded or followed by ICT (67). These studies along with the considerable toxicity associated with ICT argues against the use of ICT prior to alloBMT. A prospective randomized study is evaluating HMA vs ICT for MDS prior to alloBMT (NCT01812252). CPX-351 is also being studied in the pre-alloBMT setting for HR-MDS (NCT03572764, NCT04061239). Another trial is being conducted to evaluate immediate alloBMT vs alloBMT after 1-2 cycles of CPX-351 in patients with higher risk MDS (NCT04526288).

### F. Timing of Transplant

The timing of alloBMT is a critical consideration during the initial transplant evaluation. Delaying transplant can lead to disease progression and development of other comorbidities that preclude transplant at a later time. In contrast, premature transplant can lead to transplant related morbidity and mortality in patients who could have otherwise enjoyed a prolonged period of good quality of life without transplant.

In 2004, Cutler et al. performed a Markov model analysis using CIBMTR data to compare three transplantation strategies: transplantation at diagnosis, transplantation at leukemic progression, and transplantation at an interval prior to leukemic progression. They reported that for patients with low and intermediate-1 MDS as per the IPSS score, a delayed transplantation strategy yielded optimal survival. For patients with intermediate-2 and high risk IPSS, maximal survival was observed with transplantation at diagnosis (68). In 2014, Alessandrino et al. similarly used a Markov model to compare best available therapy versus transplant in a cohort of 1137 MDS patients. Optimal survival was observed when transplant was performed early in patients with intermediate-2 and high risk disease by IPSS, though patients with low or intermediate-1 MDS benefited from delayed transplant (69). In a European cohort of 1728 MDS patients, Della Porta et al. also applied continuous-time multistate Markov modeling and found that delaying transplant until the IPSS-R score reached intermediate resulted in a gain of life expectancy of 5.3, 4.7 and 2.8 years for patients aged ≤55, 60 and 65 years, respectively. Survival subsequently decreased with delaying transplant further (65). In summary, for patients with an indication for transplant, the preferred timing to optimize survival is early transplant as soon as a donor is available.

### G. Conditioning for Transplant

Myeloablative conditioning (MAC) alloBMT is a well-established, potentially curative therapy for MDS. However, MAC is associated with high toxicity and risk of TRM and, thus, is not feasible in most MDS patients who are commonly elderly and unfit. RIC has emerged as an appealing modality for such patients. Several retrospective studies have shown a higher risk of relapse and lower TRM with RIC compared to MAC, though mixed results regarding OS with RIC when compared with MAC (70–73).

In a randomized phase 3 trial (BMT-CTN0901), Scott et al. compared MAC (n=135) to RIC (n=137) in patients between 18 and 65 years of age with MDS or AML undergoing alloBMT (74). There was a trend towards improved OS in the MAC group, and the lack of statistical significance was attributed to the trial being stopped prematurely after 272 patients due to ethical concerns related to higher rates of relapse in the RIC arm. Long term data from this study was recently reported (75). At a median follow up of 51 months, there were higher rates of TRM in the MAC arm (HR 2.0; 95% CI 1.06 to 3.70, p=0.03), but this was counterbalanced by high rates of relapse in the RIC arm (HR 4.06; 95% CI 2.59 to 6.35; P < 0.001). MAC led to improved OS (HR 1.54; 95% CI 1.07 to 2.2; P = 0.03) and RFS (HR 2.06; 95% CI 1.48 to 2.85; p<0.001) in the entire cohort. In an analysis of only the MDS cohort, there were no statistically significant differences in TRM or relapse, though the authors concluded this was due to limited power. A similar study through the EBMT compared MAC versus RIC in 129 patients with MDS between the ages of 18-65. In contrast to the BMTCTN study, the EBMT trial showed no difference in OS or DFS based on conditioning (76). The discrepancy in the results may be due to the inclusion of AML patients in BMTCTN 0901, or potentially due to differences in the specific conditioning chemotherapy regimens used in the USA versus Europe. In a retrospective study by Bejanyan et al., RIC and MAC were compared in 4387 MDS/AML patients between 40 to 65 years stratified by DRI (77). They found that in patients with low or intermediate DRI, RIC was associated with lower TRM (HR 0.74, p<0.001) but led to greater risk of relapse (HR 1.54; p<0.001), resulting in worse DFS (HR 1.19; p=0.001). In the high or very high DRI patients, RIC trended towards lower TRM (HR 0.83, p=0.051) but led to significantly higher risk of relapse (HR 1.23; p=0.002) and ultimately similar DFS.

A significant weakness of most studies evaluating conditioning intensity is that multiple specific regimens are included in each group. This leads to uncertainty about the effects of conditioning intensity versus the potential that some specific conditioning regimens may be more effective at eliminating MDS, even at a lower intensity. To assess this, Oran et al. compared the two most commonly used RIC regimens, fludarabine with busulfan (FluBu) and fludarabine with melphalan (FluMel) in 1045 MDS patients greater than equal to 60 years through CIBMTR (78). They found that FluMel was associated with a reduced incidence of relapse (26% vs. 44% (p ≤ 0.0001). However, FluMel was also associated with greater TRM compared to FluBu (26% vs. 16%, p ≤ 0.0001). The improvement in relapse outweighed the increase in TRM in the FluMel cohort; hence, DFS was better with FluMel (48% vs. 40% at 1 year, p=0.02, and 35% vs. 27% at 3 years, p=0.01) (78).

In our practice, MAC is preferred in young, fit patients where the risk of TRM is acceptable in order to minimize the risk of relapse and maximize cure. However, the majority of patients with MDS cannot tolerate MAC. For these patients, our preferred RIC regimen is Flu/Mel, though additional studies are warranted to identify the best RIC regimen for MDS.

### H. Post-Transplant Maintenance Strategies

Relapse remains the primary driver of treatment failure after alloBMT for MDS. Hence, there is significant interest in identifying novel strategies to reduce relapse using post-transplant maintenance therapy. In the open-label, phase II, RELAZA-2 study, azacitidine was studied as a maintenance strategy for MDS/AML patients with MRD (minimal residual disease) positive status post-transplant (n=28) and post remission with ICT (n=13). They observed a delay in hematological relapse by a median of 320 days in minimal residual disease (MRD) positive patients treated with azacitidine (79). Oral azacitidine (CC-486) was studied in a phase I/II trial of 26 AML and 4 MDS patients as maintenance post alloBMT in both a 7-day and 14-day regimen resulting in 1-year DFS rates of 54% and 72%, respectively and 1-year survival rates of 86% and 81%, respectively (80). Despite these promising results, a recent phase III trial comparing post-transplant azacitidine maintenance to standard care in 187 patients showed that the median RFS and OS were not significantly different between the two groups (RFS 2.07 years in the azacitidine group versus 1.28 years in the control group; P = .43; OS 2.52 years vs 2.56 years in the azacitidine and control groups; P = .85). However, while the trial design included 12 cycles of maintenance therapy, the median number of azacitidine cycles actually received was only 4 due to toxicity, logistical reasons, and patient preferences (81). Hence, further studies are needed to prove the role of HMA maintenance therapy in this setting. Additionally, future studies should focus on specific high risk groups who may experience more benefit from post-transplant maintenance.

A number of post-transplant maintenance trials are awaiting results. A phase III randomized, double blind study of oral azacitidine vs placebo as maintenance post alloBMT in AML or MDS is currently recruiting (NCT04173533) (82). Panobinostat as a maintenance agent was also studied in post alloBMT MDS/AML patients in a phase I/II trial and now is being studied in a phase III study as maintenance post alloBMT in high risk AML and MDS patients (NCT04326764) (83). A multicenter, phase 2, open label study evaluating APR-246, a novel p53 reactivator, in combination with azacitidine for *TP53* mutated AML or MDS post alloBMT is nearly complete with results anticipated in the coming year (NCT03931291) (84). IDH1/2 inhibitors are also being studied as maintenance strategies post-transplant (NCT03515512 and NCT03564821) (85, 86).

### I. Relapse Post-AlloBMT

Post-alloBMT relapse in MDS is the main cause of treatment failure and is linked to poor prognosis irrespective of the salvage therapy used (87, 88). Management strategies include reduction or withdrawal of immunosuppressive therapy, chemotherapy, targeted therapies, donor lymphocyte infusion and possibly a second alloBMT. However, a second transplant comes with a risk of greater TRM and morbidity. A study of 2632 second alloBMT for hematologic malignancies found that overall only 20% of patients were alive at 5 years and only 15% of patients were alive and relapse free at 5 years. Outcomes in MDS patients were similar to the entire cohort (89). Patients with a longer period of remission after the first transplant were more likely to benefit from second transplant.

The combination of azacitidine and DLI for post-transplant relapse is of high interest as azacitidine not only is active against MDS, but also increases the immunogenicity of blasts and has an immunomodulatory effect that may be synergistic with DLI. In a study on 154 patients, Schroeder et al. studied azacitidine with or without DLI in patients with AML or MDS and reported an ORR of 33% with 27% CR rate. DLI was administered to 105 patients. The analysis of the entire cohort showed that diagnosis of MDS and BM blasts <13% was associated with a better OS. Two-year survival in MDS patients was 66%, which was higher than the AML cohort (29%, p=0.001) (90). Other smaller studies have shown similar results with reported 33-60% CR rates in the post alloBMT relapse setting (91, 92).

Given the poor outcomes and unclear best approach to manage post-alloBMT relapse, these patients are best served by a clinical trial. Novel cellular therapies such as chimeric antigen receptor T cells (CAR-T cells) offer promise for management of post-BMT relapse (NCT03927261). PRGN-3006 UltraCAR-T cells are currently being evaluated in this Phase 1/1b first-in-human dose escalation/dose expansion clinical trial. The study is in the dose escalation phase and has cleared the lower dose level. The study investigators previously demonstrated successful manufacturing of UltraCAR-T cells and feasibility of this approach (93).

## Special Considerations

### A. Therapy Related MDS

Therapy related MDS (t-MDS) is a category of MDS which develops in patients who have undergone therapy with antimetabolites, alkylating agents and/or radiation therapy for prior cancers (94). These therapies provoke dysplastic clonal expansion and cytogenetic abnormalities, often with complex cytogenetics and p53 mutation, leading to an aggressive form of MDS. Similarly, topoisomerase inhibitors also lead to therapy related myeloid neoplasms but they most commonly present with therapy related AML (t-AML) after possibly quickly progressing through a MDS phase (95). Due to use of myeloablative conditioning prior to autologous stem cell transplantation (autoBMT), t-MDS is detected in patients that undergo autoBMT at a range from 7% to 20% at 10 and 20 years respectively (96). t-MDS accounts for about 7-12% of MDS (97–99). In 2009, in a CIBMTR study on patients transplanted between 1990 and 2004, Litzow et al. reported a 5-year OS and DFS of 22% and 21%, respectively (100). In a cohort of 30 patients with t-MDS, Metafuni et al. reported a 43% long-term OS (101). In that study, 72.2% of patients maintained CR post alloBMT. Finke et al. reported long-term data on patients that underwent alloBMT for therapy related myeloid neoplasms and found that TRM and relapse rate were 32% and 44%, respectively, at 10 years. DFS and OS was 24% at 10 years (102). In a recent study, Metheny et al. reported a 5-year OS and DFS of 27% and 19% respectively (98).

Because the prognosis of t-MDS is unique from other forms of MDS, Quintas-Cardama et al. proposed a prognostic model specifically for patients with t-MDS called the t-MDS prognostic scoring system (TPSS) (103). They found that characteristics that affected OS and LFS were age >65 years (HR 1.63), ECOG 2-4 (HR 1.86), poor cytogenetics (HR 2.47), WHO MDS subtype ARs or RAEB-1/2 (HR 1.92), hemoglobin <11 g/dL (HR 2.24), platelets <50x10^9^/dL (HR 2.01), and transfusion dependency (HR 1.59) Zeidan et al. in a large study on 1950 MDS patients, compared the performances of IPSS, IPSS-R, MDAPSS, WPSS in patients with t-MDS (n=370, 19%). They showed that the median survival for patients with t-MDS was significantly shorter than the risk models would predict for *de novo* MDS (19 vs 46 months, p<0.005) (104). Hence, transplant should be considered for these patients even the setting of lower risk disease by standard prediction models.

### B. Hypoplastic MDS

A subset of MDS characterized by low BM cellularity, usually less than 25%, is called hypoplastic MDS and compromises approximately 10-15% of MDS cases (105). This poses a diagnostic challenge for physicians and pathologists due to difficulty distinguishing from aplastic anemia. These patients are typically treated with immunosuppression and potentially alloBMT (106). There is limited data on alloBMT in patients with hypoplastic MDS. Since most patients with hypoplastic MDS have low risk MDS, data from low risk MDS is extrapolated to hypoplastic MDS. Patients with hypoplastic MDS share similar disease biology to aplastic anemia (AA) and alloBMT has shown excellent responses in patients with AA (107, 108).

### C. MDS Originating From Germ Line Mutations

Implications for patients with an inherited predisposition for MDS are mainly related to donor selection. Though data is limited, transplant from a donor with the same genetic pre-disposition to MDS could theoretically increase the risk of a donor-derived MDS after transplant. Thus, potential related donors should undergo genetic testing to rule out the genetic syndrome under consideration.

For the patient, early detection and timely alloBMT prior to progression to leukemia is essential. Inherited bone marrow failure syndromes may carry a risk of increased toxicity from the conditioning regimen and hence might require RIC regimens (109). Outcomes of transplant for MDS originating from germline mutations were reported by Lindsley et al. (52). *GATA2, PIGA* and compound heterozygous mutations in the Shwachman–Diamond syndrome–associated *SBDS* gene were significantly more common in younger adults (<40 years old). *GATA2* and *PIGA* mutations were associated with better prognosis, while patients with *SBDS* gene had shorter survival (1.2 years vs not reached; p=0.009).

## Future Perspectives

With two recent prospective trials confirming an OS benefit with alloBMT for patients with HR-MDS, the role of transplant for MDS should increase in the coming years (27, 28). Thus, a strong focus on trials to optimize alloBMT for MDS should remain a priority in the transplant community. Most importantly, relapse rates remain high after alloBMT so trials should focus on relapse reduction. Maintenance therapies, though not yet proven, are an exciting approach to preventing relapse with multiple ongoing trials. The current focus is largely on maintenance drug therapies, but future trials may also explore the role of adoptive cellular therapies given after alloBMT to boost the graft-versus-tumor effect. Improvement in conditioning regimens may also yield improved relapse rates. While most members of the transplant community favor more intensive conditioning for fit patients, concomitant increases in TRM limit the potential for survival benefit. More granular studies that identify specific regimens that are effective for MDS may help improve the balance of effectiveness and toxicity. Further, if maintenance therapies prove effective, the need for intensive conditioning could be mitigated. For those patients who do experience relapse, studies defining the best management approach are warranted. Further, because outcomes are typically poor, novel therapies including targeted or cellular therapies for post-transplant relapse merit investigation.

In regards to prognostication, studies identifying the role of new prognostic models and somatic mutations, such as the new personalized prediction model by Nazha et al., on alloBMT are warranted (25). Specifically, the implementation of these models for identifying patients in need of transplant is not well defined. Determining the prognostic impact of these models on post-transplant outcomes would help patients make more informed decisions regarding pursuing transplant. Further, clarifying the role of treatment to correct negative prognostic factors, such as clearing high risk mutations before transplant, will help determine successful approaches to pre-transplant therapy in the modern era. Similarly, as age is increasingly deemphasized as a metric for transplant candidacy, robust metrics for evaluating patient fitness for transplant are needed to better identify suitable transplant candidates.

Finally, it must be noted that the role and indications for transplant are dynamic and may change over time. While alloBMT provides superior survival over current therapies, emerging non-transplant therapies could eventually provide similar or superior disease control with less toxicity.

## Conclusions

Despite recent advances, alloBMT remains the only curative treatment for MDS. However since alloBMT is a procedure with a high risk of morbidity and mortality, the patients that are taken to transplant must be chosen carefully. Both disease and patient related factors are important when considering alloBMT for each patient. Based on prospective studies, alloBMT is clearly indicated and provides a survival benefit for patients with HR-MDS by traditional scoring systems. While less clearly defined, patients with LR-MDS by traditional scoring systems should still be offered transplant in cases of high-risk features such as high transfusion burden, severe leukopenia, high risk somatic mutations, significant fibrosis, or HMA failure. Because MDS largely affects older adults, many patients with an indication for transplant could not receive transplant historically. Modern approaches to transplant including RIC regimens and alternative donors have broadened eligibility and confirmed a survival benefit even in patients over 70 years of age. Rather than age, eligibility for transplant should be determined based on functional status, comorbidities, frailty, and a careful discussion of the pros/cons of transplant in the context of the patient’s goals of care. In general, when transplant is indicated, early transplant offers superior outcomes versus delaying transplant. Thus, all patients that are potentially transplant-eligible should be referred for transplant consultation as soon as the diagnosis is made. Interventions post alloBMT are dependent on individual relapse risk and maintenance strategies are being studied to minimize relapse.

## Author Contributions

AGJ wrote the manuscript. HE thoroughly revised and edited the manuscript. Both authors have made substantial, direct and intellectual contribution to the work, and approved it for publication.

## Conflict of Interest

The authors declare that the research was conducted in the absence of any commercial or financial relationships that could be construed as a potential conflict of interest.

## Publisher’s Note

All claims expressed in this article are solely those of the authors and do not necessarily represent those of their affiliated organizations, or those of the publisher, the editors and the reviewers. Any product that may be evaluated in this article, or claim that may be made by its manufacturer, is not guaranteed or endorsed by the publisher.

## References

[1] Arber DA Orazi A Hasserjian R Thiele J Borowitz MJ Le Beau MM. The 2016 Revision to the World Health Organization Classification of Myeloid Neoplasms and Acute Leukemia. Blood (2016) 127(20):2391–405. doi: 10.1182/blood-2016-03-643544 27069254

[2] Zeidan AM Shallis RM Wang R Davidoff A Ma X. Epidemiology of Myelodysplastic Syndromes: Why Characterizing the Beast Is a Prerequisite to Taming it. Blood Rev (2019) 34:1–15. doi: 10.1016/j.blre.2018.09.001 30314642

[3] Nachtkamp K Stark R Strupp C Kündgen A Giagounidis A Aul C. Causes of Death in 2877 Patients With Myelodysplastic Syndromes. Ann Hematol (2016) 95(6):937–44. doi: 10.1007/s00277-016-2649-3 27025507

[4] Fenaux P Mufti GJ Hellstrom-Lindberg E Santini V Finelli C Giagounidis A. Efficacy of Azacitidine Compared With That of Conventional Care Regimens in the Treatment of Higher-Risk Myelodysplastic Syndromes: A Randomised, Open-Label, Phase III Study. Lancet Oncol (2009) 10(3):223–32. doi: 10.1016/S1470-2045(09)70003-8 PMC4086808 19230772

[5] de Witte T Bowen D Robin M Malcovati L Niederwieser D Yakoub-Agha I. Allogeneic Hematopoietic Stem Cell Transplantation for MDS and CMML: Recommendations From an International Expert Panel. Blood (2017) 129(13):1753–62. doi: 10.1182/blood-2016-06-724500 PMC5524528 28096091

[6] Tanaka TN Bejar R. MDS Overlap Disorders and Diagnostic Boundaries. Blood (2019) 133(10):1086–95. doi: 10.1182/blood-2018-10-844670 30670443

[7] Shallis RM Zeidan AM. Management of the Older Patient With Myelodysplastic Syndrome. Drugs Aging (2021) 38(9):751–67. doi: 10.1007/s40266-021-00881-3 34342860

[8] Cheson BD Greenberg PL Bennett JM Lowenberg B Wijermans PW Nimer SD. Clinical Application and Proposal for Modification of the International Working Group (IWG) Response Criteria in Myelodysplasia. Blood (2006) 108(2):419–25. doi: 10.1182/blood-2005-10-4149 16609072

[9] Haase D Germing U Schanz J Pfeilstöcker M Nösslinger T Hildebrandt B. New Insights Into the Prognostic Impact of the Karyotype in MDS and Correlation With Subtypes: Evidence From a Core Dataset of 2124 Patients. Blood (2007) 110(13):4385–95. doi: 10.1182/blood-2007-03-082404 17726160

[10] Schanz J Tüchler H Solé F Mallo M Luño E Cervera J. New Comprehensive Cytogenetic Scoring System for Primary Myelodysplastic Syndromes (MDS) and Oligoblastic Acute Myeloid Leukemia After MDS Derived From an International Database Merge. J Clin Oncol (2012) 30(8):820–9. doi: 10.1200/JCO.2011.35.6394 PMC4874200 22331955

[11] Shallis RM Ahmad R Zeidan AM. The Genetic and Molecular Pathogenesis of Myelodysplastic Syndromes. Eur J Haematol (2018) 101(3):260–71. doi: 10.1111/ejh.13092 29742289

[12] Haferlach T Nagata Y Grossmann V Okuno Y Bacher U Nagae G. Landscape of Genetic Lesions in 944 Patients With Myelodysplastic Syndromes. Leukemia (2014) 28(2):241–7. doi: 10.1038/leu.2013.336 PMC3918868 24220272

[13] Papaemmanuil E Gerstung M Malcovati L Tauro S Gundem G Van Loo P. Clinical and Biological Implications of Driver Mutations in Myelodysplastic Syndromes. Blood (2013) 122(22):3616–27. doi: 10.1182/blood-2013-08-518886 PMC3837510 24030381

[14] Bejar R Stevenson K Abdel-Wahab O Galili N Nilsson B Garcia-Manero G. Clinical Effect of Point Mutations in Myelodysplastic Syndromes. N Engl J Med (2011) 364(26):2496–506. doi: 10.1056/NEJMoa1013343 PMC3159042 21714648

[15] Palomo L Meggendorfer M Hutter S Twardziok S Ademà V Fuhrmann I. Molecular Landscape and Clonal Architecture of Adult Myelodysplastic/Myeloproliferative Neoplasms. Blood (2020) 136(16):1851–62. doi: 10.1182/blood.2019004229 PMC7645608 32573691

[16] Heuser M Yun H Thol F. Epigenetics in Myelodysplastic Syndromes. Semin Cancer Biol (2018) 51:170–9. doi: 10.1016/j.semcancer.2017.07.009 PMC7116652 28778402

[17] Bejar R Stevenson KE Caughey B Lindsley RC Mar BG Stojanov P. Somatic Mutations Predict Poor Outcome in Patients With Myelodysplastic Syndrome After Hematopoietic Stem-Cell Transplantation. J Clin Oncol (2014) 32(25):2691–8. doi: 10.1200/JCO.2013.52.3381 PMC4207878 25092778

[18] Haferlach T. The Molecular Pathology of Myelodysplastic Syndrome. Pathobiology (2019) 86(1):24–9. doi: 10.1159/000488712 29791902

[19] Lodé L Ménard A Flet L Richebourg S Loirat M Eveillard M. Emergence and Evolution of. Haematologica (2018) 103(4):e143–6. doi: 10.3324/haematol.2017.181404 PMC5865441 29269520

[20] Jädersten M Saft L Smith A Kulasekararaj A Pomplun S Göhring G. TP53 Mutations in Low-Risk Myelodysplastic Syndromes With Del(5q) Predict Disease Progression. J Clin Oncol (2011) 29(15):1971–9. doi: 10.1200/JCO.2010.31.8576 21519010

[21] National Comprehensive Cancer Network. Myelodysplastic Syndromes (Version 1.2022). Available at: https://www.nccn.org/professionals/physician_gls/pdf/mds.pdf (Accessed October 30, 2021).

[22] Greenberg PL Stone RM Al-Kali A Barta SK Bejar R Bennett JM. Myelodysplastic Syndromes, Version 2.2017, NCCN Clinical Practice Guidelines in Oncology. J Natl Compr Canc Netw (2017) 15(1):60–87. doi: 10.6004/jnccn.2017.0007 28040720

[23] Greenberg P Cox C LeBeau MM Fenaux P Morel P Sanz G. International Scoring System for Evaluating Prognosis in Myelodysplastic Syndromes. Blood (1997) 89(6):2079–88. doi: 10.1182/blood.V89.6.2079 9058730

[24] Greenberg PL Tuechler H Schanz J Sanz G Garcia-Manero G Solé F. Revised International Prognostic Scoring System for Myelodysplastic Syndromes. Blood (2012) 120(12):2454–65. doi: 10.1182/blood-2012-03-420489 PMC4425443 22740453

[25] Nazha A Komrokji R Meggendorfer M Jia X Radakovich N Shreve J. Personalized Prediction Model to Risk Stratify Patients With Myelodysplastic Syndromes. J Clin Oncol (2021) 39(33):3737–46. doi: 10.1200/JCO.20.02810 PMC8601291 34406850

[26] Robin M Porcher R Adès L Raffoux E Michallet M François S. HLA-Matched Allogeneic Stem Cell Transplantation Improves Outcome of Higher Risk Myelodysplastic Syndrome A Prospective Study on Behalf of SFGM-TC and GFM. Leukemia (2015) 29(7):1496–501. doi: 10.1038/leu.2015.37 25676424

[27] Nakamura R Saber W Martens MJ Ramirez A Scott B Oran B. Biologic Assignment Trial of Reduced-Intensity Hematopoietic Cell Transplantation Based on Donor Availability in Patients 50-75 Years of Age With Advanced Myelodysplastic Syndrome. J Clin Oncol (2021) 39(30):3328–39. doi: 10.1200/JCO.20.03380 PMC8791814 34106753

[28] Kroger N Sockel K Wolschke C Bethge W Schlenk RF Wolf D. Comparison Between 5-Azacytidine Treatment and Allogeneic Stem-Cell Transplantation in Elderly Patients With Advanced MDS According to Donor Availability (VidazaAllo Study). J Clin Oncol (2021) 39(30):3318–27. doi: 10.1200/JCO.20.02724 34283629

[29] Killick SB Ingram W Culligan D Enright H Kell J Payne EM. British Society for Haematology Guidelines for the Management of Adult Myelodysplastic Syndromes. Br J Haematol (2021) 194(2):267–81. doi: 10.1111/bjh.17612 34180045

[30] Prébet T Gore SD Esterni B Gardin C Itzykson R Thepot S. Outcome of High-Risk Myelodysplastic Syndrome After Azacitidine Treatment Failure. J Clin Oncol (2011) 29(24):3322–7. doi: 10.1200/JCO.2011.35.8135 PMC4859209 21788559

[31] Jabbour E Garcia-Manero G Batty N Shan J O’Brien S Cortes J. Outcome of Patients With Myelodysplastic Syndrome After Failure of Decitabine Therapy. Cancer (2010) 116(16):3830–4. doi: 10.1002/cncr.25247 PMC4295788 20564137

[32] Koenecke C Göhring G de Wreede LC van Biezen A Scheid C Volin L. Impact of the Revised International Prognostic Scoring System, Cytogenetics and Monosomal Karyotype on Outcome After Allogeneic Stem Cell Transplantation for Myelodysplastic Syndromes and Secondary Acute Myeloid Leukemia Evolving From Myelodysplastic Syndromes: A Retrospective Multicenter Study of the European Society of Blood and Marrow Transplantation. Haematologica (2015) 100(3):400–8. doi: 10.3324/haematol.2014.116715 PMC4349280 25552702

[33] Pease DF Ross JA Poynter JN Nguyen PL Hirsch B Cioc A. Differences in Community and Academic Practice Patterns for Newly Diagnosed Myelodysplastic Syndromes (MDS) Patients. Cancer Epidemiol (2015) 39(2):222–8. doi: 10.1016/j.canep.2015.01.006 PMC4361261 25701277

[34] Platzbecker U Schetelig J Finke J Trenschel R Scott BL Kobbe G. Allogeneic Hematopoietic Cell Transplantation in Patients Age 60-70 Years With *De Novo* High-Risk Myelodysplastic Syndrome or Secondary Acute Myelogenous Leukemia: Comparison With Patients Lacking Donors Who Received Azacitidine. Biol Blood Marrow Transplant (2012) 18(9):1415–21. doi: 10.1016/j.bbmt.2012.05.003 PMC4560484 22579634

[35] McClune BL Weisdorf DJ Pedersen TL Tunes da Silva G Tallman MS Sierra J. Effect of Age on Outcome of Reduced-Intensity Hematopoietic Cell Transplantation for Older Patients With Acute Myeloid Leukemia in First Complete Remission or With Myelodysplastic Syndrome. J Clin Oncol (2010) 28(11):1878–87. doi: 10.1200/JCO.2009.25.4821 PMC2860368 20212255

[36] Lim Z Brand R Martino R van Biezen A Finke J Bacigalupo A. Allogeneic Hematopoietic Stem-Cell Transplantation for Patients 50 Years or Older With Myelodysplastic Syndromes or Secondary Acute Myeloid Leukemia. J Clin Oncol (2010) 28(3):405–11. doi: 10.1200/JCO.2009.21.8073 20008642

[37] Rockwood K Song X MacKnight C Bergman H Hogan DB McDowell I. A Global Clinical Measure of Fitness and Frailty in Elderly People. CMAJ (2005) 173(5):489–95. doi: 10.1503/cmaj.050051 PMC1188185 16129869

[38] Sakatoku K Takeoka Y Miura A Araki T Fujitani Y Yamamura R. Combination of Frailty Status and Comorbidity Score Improves the Stratification of Survival in Patients With Myelodysplastic Syndrome Owing to Good Predictive Capability for Infection-Related Mortality. Clin Lymphoma Myeloma Leuk (2019) 19(12):799–805. doi: 10.1016/j.clml.2019.09.610 31648956

[39] Sorror ML Storb RF Sandmaier BM Maziarz RT Pulsipher MA Maris MB. Comorbidity-Age Index: A Clinical Measure of Biologic Age Before Allogeneic Hematopoietic Cell Transplantation. J Clin Oncol (2014) 32(29):3249–56. doi: 10.1200/JCO.2013.53.8157 PMC4178523 25154831

[40] Sorror ML Sandmaier BM Storer BE Maris MB Baron F Maloney DG. Comorbidity and Disease Status Based Risk Stratification of Outcomes Among Patients With Acute Myeloid Leukemia or Myelodysplasia Receiving Allogeneic Hematopoietic Cell Transplantation. J Clin Oncol (2007) 25(27):4246–54. doi: 10.1200/JCO.2006.09.7865 17724349

[41] Artz A Sorror M. Available at: https://web.emmes.com/study/bmt2/protocol/1704_protocol/1704_protocol.html https://web.emmes.com/study/bmt2/protocol/1704_protocol/1704_protocol.html2021.

[42] Abel GA Buckstein R. Integrating Frailty, Comorbidity, and Quality of Life in the Management of Myelodysplastic Syndromes. Am Soc Clin Oncol Educ Book (2016) 35:e337–44. doi: 10.1200/EDBK_158639 27249740

[43] Della Porta MG Alessandrino EP Bacigalupo A van Lint MT Malcovati L Pascutto C. Predictive Factors for the Outcome of Allogeneic Transplantation in Patients With MDS Stratified According to the Revised IPSS-R. Blood (2014) 123(15):2333–42. doi: 10.1182/blood-2013-12-542720 24558201

[44] Scheid C de Wreede L van Biezen A Koenecke C Göhring G Volin L. Validation of the Revised IPSS at Transplant in Patients With Myelodysplastic Syndrome/Transformed Acute Myelogenous Leukemia Receiving Allogeneic Stem Cell Transplantation: A Retrospective Analysis of the EBMT Chronic Malignancies Working Party. Bone Marrow Transplant (2017) 52(11):1519–25. doi: 10.1038/bmt.2017.171 PMC5671928 28892084

[45] Della Porta MG Malcovati L. Myelodysplastic Syndromes With Bone Marrow Fibrosis. Haematologica (2011) 96(2):180–3. doi: 10.3324/haematol.2010.039875 PMC3031682 21282718

[46] Kröger N Zabelina T van Biezen A Brand R Niederwieser D Martino R. Allogeneic Stem Cell Transplantation for Myelodysplastic Syndromes With Bone Marrow Fibrosis. Haematologica (2011) 96(2):291–7. doi: 10.3324/haematol.2010.031229 PMC3031698 20971823

[47] Alessandrino EP Della Porta MG Bacigalupo A Van Lint MT Falda M Onida F. WHO Classification and WPSS Predict Posttransplantation Outcome in Patients With Myelodysplastic Syndrome: A Study From the Gruppo Italiano Trapianto Di Midollo Osseo (GITMO). Blood (2008) 112(3):895–902. doi: 10.1182/blood-2008-03-143735 18497321

[48] Armand P Gibson CJ Cutler C Ho VT Koreth J Alyea EP. A Disease Risk Index for Patients Undergoing Allogeneic Stem Cell Transplantation. Blood (2012) 120(4):905–13. doi: 10.1182/blood-2012-03-418202 PMC3412351 22709687

[49] Armand P Kim HT Logan BR Wang Z Alyea EP Kalaycio ME. Validation and Refinement of the Disease Risk Index for Allogeneic Stem Cell Transplantation. Blood (2014) 123(23):3664–71. doi: 10.1182/blood-2014-01-552984 PMC4047501 24744269

[50] Fernández-Caballero M Jiménez Lorenzo MJ Morgades de la Fe M Ferrà Coll C Vives Polo S Abril Sabater L. Impact of Risk Scores in Outcome of Patients With Myeloid Neoplasms After Allogeneic Stem Cell Transplant. Med Clin (Barc) (2021) S0025-7753(21):00430-9. doi: 10.1016/j.medcli.2021.05.025 34404519

[51] Della Porta MG Gallì A Bacigalupo A Zibellini S Bernardi M Rizzo E. Clinical Effects of Driver Somatic Mutations on the Outcomes of Patients With Myelodysplastic Syndromes Treated With Allogeneic Hematopoietic Stem-Cell Transplantation. J Clin Oncol (2016) 34(30):3627–37. doi: 10.1200/JCO.2016.67.3616 PMC6366344 27601546

[52] Lindsley RC Saber W Mar BG Redd R Wang T Haagenson MD. Prognostic Mutations in Myelodysplastic Syndrome After Stem-Cell Transplantation. N Engl J Med (2017) 376(6):536–47. doi: 10.1056/NEJMoa1611604 PMC5438571 28177873

[53] Bernard E Nannya Y Hasserjian RP Devlin SM Tuechler H Medina-Martinez JS. Implications of TP53 Allelic State for Genome Stability, Clinical Presentation and Outcomes in Myelodysplastic Syndromes. Nat Med (2020) 26(10):1549–56. doi: 10.1038/s41591-020-1008-z PMC8381722 32747829

[54] Novak P Zabelina T Wolschke C Ayuk F Christopeit M Kröger N. Allogeneic Stem Cell Transplantation for Patients With Lower-Risk Myelodysplastic Syndrome. Biol Blood Marrow Transplant (2020) 26(11):2047–52. doi: 10.1016/j.bbmt.2020.07.018 32717435

[55] Gagelmann N Eikema DJ Stelljes M Beelen D de Wreede L Mufti G. Optimized EBMT Transplant-Specific Risk Score in Myelodysplastic Syndromes After Allogeneic Stem-Cell Transplantation. Haematologica (2019) 104(5):929–36. doi: 10.3324/haematol.2018.200808 PMC6518902 30655377

[56] del Cañizo MC Martínez C Conde E Vallejo C Brunet S Sanz G. Peripheral Blood Is Safer Than Bone Marrow as a Source of Hematopoietic Progenitors in Patients With Myelodysplastic Syndromes Who Receive an Allogeneic Transplantation. Results From the Spanish Registry. Bone Marrow Transplant (2003) 32(10):987–92. doi: 10.1038/sj.bmt.1704246 14595386

[57] Guardiola P Runde V Bacigalupo A Ruutu T Locatelli F Boogaerts MA. Retrospective Comparison of Bone Marrow and Granulocyte Colony-Stimulating Factor-Mobilized Peripheral Blood Progenitor Cells for Allogeneic Stem Cell Transplantation Using HLA Identical Sibling Donors in Myelodysplastic Syndromes. Blood (2002) 99(12):4370–8. doi: 10.1182/blood.V99.12.4370 12036864

[58] Anasetti C Logan BR Lee SJ Waller EK Weisdorf DJ Wingard JR. Peripheral-Blood Stem Cells Versus Bone Marrow From Unrelated Donors. N Engl J Med (2012) 367(16):1487–96. doi: 10.1056/NEJMoa1203517 PMC3816375 23075175

[59] Grunwald MR Zhang MJ Elmariah H Johnson MH St Martin A Bashey A. Alternative Donor Transplantation for Myelodysplastic Syndromes: Haploidentical Relative and Matched Unrelated Donors. Blood Adv (2021) 5(4):975–83. doi: 10.1182/bloodadvances.2020003654 PMC7903230 33576783

[60] Gerds AT Woo Ahn K Hu ZH Abdel-Azim H Akpek G Aljurf M. Outcomes After Umbilical Cord Blood Transplantation for Myelodysplastic Syndromes. Biol Blood Marrow Transplant (2017) 23(6):971–9. doi: 10.1016/j.bbmt.2017.03.014 PMC5474679 28288952

[61] Festuccia M Deeg HJ Gooley TA Baker K Wood BL Fang M. Minimal Identifiable Disease and the Role of Conditioning Intensity in Hematopoietic Cell Transplantation for Myelodysplastic Syndrome and Acute Myelogenous Leukemia Evolving From Myelodysplastic Syndrome. Biol Blood Marrow Transplant (2016) 22(7):1227–33. doi: 10.1016/j.bbmt.2016.03.029 PMC4999078 27064057

[62] Yahng SA Kim M Kim TM Jeon YW Yoon JH Shin SH. Better Transplant Outcome With Pre-Transplant Marrow Response After Hypomethylating Treatment in Higher-Risk MDS With Excess Blasts. Oncotarget (2017) 8(7):12342–54. doi: 10.18632/oncotarget.12511 PMC5355349 27729615

[63] Potter VT Iacobelli S van Biezen A Maertens J Bourhis JH Passweg JR. Comparison of Intensive Chemotherapy and Hypomethylating Agents Before Allogeneic Stem Cell Transplantation for Advanced Myelodysplastic Syndromes: A Study of the Myelodysplastic Syndrome Subcommittee of the Chronic Malignancies Working Party of the European Society for Blood and Marrow Transplant Research. Biol Blood Marrow Transplant (2016) 22(9):1615–20. doi: 10.1016/j.bbmt.2016.05.026 27264633

[64] Robin M Porcher R Zinke-Cerwenka W van Biezen A Volin L Mufti G. Allogeneic Haematopoietic Stem Cell Transplant in Patients With Lower Risk Myelodysplastic Syndrome: A Retrospective Analysis on Behalf of the Chronic Malignancy Working Party of the EBMT. Bone Marrow Transplant (2017) 52(7):1081. doi: 10.1038/bmt.2017.86 28677682

[65] Della Porta MG Jackson CH Alessandrino EP Rossi M Bacigalupo A van Lint MT. Decision Analysis of Allogeneic Hematopoietic Stem Cell Transplantation for Patients With Myelodysplastic Syndrome Stratified According to the Revised International Prognostic Scoring System. Leukemia (2017) 31(11):2449–57. doi: 10.1038/leu.2017.88 PMC6086331 28321120

[66] Gerds AT Gooley TA Estey EH Appelbaum FR Deeg HJ Scott BL. Pretransplantation Therapy With Azacitidine vs Induction Chemotherapy and Posttransplantation Outcome in Patients With MDS. Biol Blood Marrow Transplant (2012) 18(8):1211–8. doi: 10.1016/j.bbmt.2012.01.009 PMC3376402 22252125

[67] Damaj G Duhamel A Robin M Beguin Y Michallet M Mohty M. Impact of Azacitidine Before Allogeneic Stem-Cell Transplantation for Myelodysplastic Syndromes: A Study by the Société Française De Greffe De Moelle Et De Thérapie-Cellulaire and the Groupe-Francophone Des Myélodysplasies. J Clin Oncol (2012) 30(36):4533–40. doi: 10.1200/JCO.2012.44.3499 23109707

[68] Cutler CS Lee SJ Greenberg P Deeg HJ Pérez WS Anasetti C. A Decision Analysis of Allogeneic Bone Marrow Transplantation for the Myelodysplastic Syndromes: Delayed Transplantation for Low-Risk Myelodysplasia Is Associated With Improved Outcome. Blood (2004) 104(2):579–85. doi: 10.1182/blood-2004-01-0338 15039286

[69] Alessandrino EP Porta MG Malcovati L Jackson CH Pascutto C Bacigalupo A. Optimal Timing of Allogeneic Hematopoietic Stem Cell Transplantation in Patients With Myelodysplastic Syndrome. Am J Hematol (2013) 88(7):581–8. doi: 10.1002/ajh.23458 PMC3736162 23606215

[70] Aoudjhane M Labopin M Gorin NC Shimoni A Ruutu T Kolb HJ. Comparative Outcome of Reduced Intensity and Myeloablative Conditioning Regimen in HLA Identical Sibling Allogeneic Haematopoietic Stem Cell Transplantation for Patients Older Than 50 Years of Age With Acute Myeloblastic Leukaemia: A Retrospective Survey From the Acute Leukemia Working Party (ALWP) of the European Group for Blood and Marrow Transplantation (EBMT). Leukemia (2005) 19(12):2304–12. doi: 10.1038/sj.leu.2403967 16193083

[71] Scott BL Sandmaier BM Storer B Maris MB Sorror ML Maloney DG. Myeloablative vs Nonmyeloablative Allogeneic Transplantation for Patients With Myelodysplastic Syndrome or Acute Myelogenous Leukemia With Multilineage Dysplasia: A Retrospective Analysis. Leukemia (2006) 20(1):128–35. doi: 10.1038/sj.leu.2404010 16270037

[72] Shimoni A Hardan I Shem-Tov N Yerushalmi R Nagler A. Allogeneic Hematopoietic Stem-Cell Transplantation in AML and MDS Using Myeloablative Versus Reduced-Intensity Conditioning: Long-Term Follow-Up. Leukemia (2010) 24(5):1050–2. doi: 10.1038/leu.2010.12 20147978

[73] Luger SM Ringdén O Zhang MJ Pérez WS Bishop MR Bornhauser M. Similar Outcomes Using Myeloablative vs Reduced-Intensity Allogeneic Transplant Preparative Regimens for AML or MDS. Bone Marrow Transplant (2012) 47(2):203–11. doi: 10.1038/bmt.2011.69 PMC3134582 21441963

[74] Scott BL Pasquini MC Logan BR Wu J Devine SM Porter DL. Myeloablative Versus Reduced-Intensity Hematopoietic Cell Transplantation for Acute Myeloid Leukemia and Myelodysplastic Syndromes. J Clin Oncol (2017) 35(11):1154–61. doi: 10.1200/JCO.2016.70.7091 PMC5455603 28380315

[75] Scott BL Pasquini MC Fei M Fraser R Wu J Devine SM. Myeloablative Versus Reduced-Intensity Conditioning for Hematopoietic Cell Transplantation in Acute Myelogenous Leukemia and Myelodysplastic Syndromes-Long-Term Follow-Up of the BMT CTN 0901 Clinical Trial. Transplant Cell Ther (2021) 27(6):483.e1–.e6. doi: 10.1016/j.jtct.2021.02.031 PMC8217373 33775615

[76] Kroger N Iacobelli S Franke G-N Platzbecker U Uddin R Hubel K. Dose-Reduced Versus Standard Conditioning Followed by Allogeneic Stem-Cell Transplantation for Patients With Myelodysplastic Syndrome: A Prospective Randomized Phase III Study of the EBMT (RICMAC Trial). JCO (2017) 35(19):2157–64. doi: 10.1200/JCO.2016.70.7349 28463633

[77] Bejanyan N Zhang M Bo-Subait K Brunstein C Wang H Warlick ED. Myeloablative Conditioning for Allogeneic Transplantation Results in Superior Disease-Free Survival for Acute Myelogenous Leukemia and Myelodysplastic Syndromes With Low/Intermediate But Not High Disease Risk Index: A Center for International Blood and Marrow Transplant Research Study. Transplant Cell Ther (2021) 27(1):68.e1–9. doi: 10.1016/j.bbmt.2020.09.026 PMC8015679 33010430

[78] Oran B Ahn KW Fretham C Beitinjaneh A Bashey A Pawarode A. Fludarabine and Melphalan Compared With Reduced Doses of Busulfan and Fludarabine Improves Transplant Outcomes in Older MDS Patients. Transplant Cell Ther (2021) (11):921.e1-921.e10. doi: 10.1016/j.jtct.2021.08.007 PMC9562611 34403791

[79] Platzbecker U Middeke JM Sockel K Herbst R Wolf D Baldus CD. Measurable Residual Disease-Guided Treatment With Azacitidine to Prevent Haematological Relapse in Patients With Myelodysplastic Syndrome and Acute Myeloid Leukaemia (RELAZA2): An Open-Label, Multicentre, Phase 2 Trial. Lancet Oncol (2018) 19(12):1668–79. doi: 10.1016/S1470-2045(18)30580-1 30442503

[80] de Lima M Oran B Champlin RE Papadopoulos EB Giralt SA Scott BL. CC-486 Maintenance After Stem Cell Transplantation in Patients With Acute Myeloid Leukemia or Myelodysplastic Syndromes. Biol Blood Marrow Transplant (2018) 24(10):2017–24. doi: 10.1016/j.bbmt.2018.06.016 PMC8059405 29933073

[81] Oran B de Lima M Garcia-Manero G Thall PF Lin R Popat U. A Phase 3 Randomized Study of 5-Azacitidine Maintenance vs Observation After Transplant in High-Risk AML and MDS Patients. Blood Adv (2020) 4(21):5580–8. doi: 10.1182/bloodadvances.2020002544 PMC7656915 33170934

[82] ClinicalTrials.gov. Randomised Study of Oral Azacitidine vs Placebo Maintenance in AML or MDS Patients After Allo-SCT (AMADEUS) (2021). Available at: https://clinicaltrials.gov/ct2/show/NCT04173533.

[83] Bug G Burchert A Wagner EM Kröger N Berg T Güller S. Phase I/II Study of the Deacetylase Inhibitor Panobinostat After Allogeneic Stem Cell Transplantation in Patients With High-Risk MDS or AML (PANOBEST Trial). Leukemia (2017) 31(11):2523–5. doi: 10.1038/leu.2017.242 PMC5668491 28751769

[84] ClinicalTrials.gov. APR-246 in Combination With Azacitidine for TP53 Mutated AML (Acute Myeloid Leukemia) or MDS (Myelodysplastic Syndromes) Following Allogeneic Stem Cell Transplant (2021). Available at: https://clinicaltrials.gov/ct2/show/NCT03931291.

[85] Clinicaltrials.gov. IDH1 Inhibition Using Ivosidenib as Maintenance Therapy for IDH1-Mutant Myeloid Neoplasms Following Allogeneic Stem Cell Transplantation (2021). Available at: https://clinicaltrials.gov/ct2/show/NCT03564821.

[86] ClinicalTrials.gov. IDH2 Inhibition Using Enasidenib as Maintenance Therapy for IDH2-Mutant Myeloid Neoplasms Following Allogeneic Stem Cell Transplantation (2021). Available at: https://clinicaltrials.gov/ct2/show/NCT03515512.

[87] Bejanyan N Weisdorf DJ Logan BR Wang HL Devine SM de Lima M. Survival of Patients With Acute Myeloid Leukemia Relapsing After Allogeneic Hematopoietic Cell Transplantation: A Center for International Blood and Marrow Transplant Research Study. Biol Blood Marrow Transplant (2015) 21(3):454–9. doi: 10.1016/j.bbmt.2014.11.007 PMC4329076 25460355

[88] El-Cheikh J Massoud R Fares E Kreidieh N Mahfouz R Charafeddine M. Low-Dose 5-Azacytidine as Preventive Therapy for Relapse of AML and MDS Following Allogeneic HCT. Bone Marrow Transplant (2017) 52(6):918–21. doi: 10.1038/bmt.2017.31 28368381

[89] Ruutu T de Wreede LC van Biezen A Brand R Mohty M Dreger P. Second Allogeneic Transplantation for Relapse of Malignant Disease: Retrospective Analysis of Outcome and Predictive Factors by the EBMT. Bone Marrow Transplant (2015) 50(12):1542–50. doi: 10.1038/bmt.2015.186 26367221

[90] Schroeder T Rachlis E Bug G Stelljes M Klein S Steckel NK. Treatment of Acute Myeloid Leukemia or Myelodysplastic Syndrome Relapse After Allogeneic Stem Cell Transplantation With Azacitidine and Donor Lymphocyte Infusions–a Retrospective Multicenter Analysis From the German Cooperative Transplant Study Group. Biol Blood Marrow Transplant (2015) 21(4):653–60. doi: 10.1016/j.bbmt.2014.12.016 25540937

[91] Bolaños-Meade J Smith BD Gore SD McDevitt MA Luznik L Fuchs EJ. 5-Azacytidine as Salvage Treatment in Relapsed Myeloid Tumors After Allogeneic Bone Marrow Transplantation. Biol Blood Marrow Transplant (2011) 17(5):754–8. doi: 10.1016/j.bbmt.2010.10.008 PMC3090635 20951817

[92] Tessoulin B Delaunay J Chevallier P Loirat M Ayari S Peterlin P. Azacitidine Salvage Therapy for Relapse of Myeloid Malignancies Following Allogeneic Hematopoietic SCT. Bone Marrow Transplant (2014) 49(4):567–71. doi: 10.1038/bmt.2013.233 24488048

[93] Sallman DA Elmariah H Sweet KL Talati C Mishra A Kelley LL. A Phase 1/1b Safety Study of Prgn-3006 Ultracar-T™ in Patients With Relapsed or Refractory CD33-Positive Acute Myeloid Leukemia and Higher Risk Myelodysplastic Syndrome. Blood (2020) 136:17. doi: 10.1182/blood-2020-139417

[94] Bhatia S. Therapy-Related Myelodysplasia and Acute Myeloid Leukemia. Semin Oncol (2013) 40(6):666–75. doi: 10.1053/j.seminoncol.2013.09.013 PMC3867743 24331189

[95] Godley LA Larson RA. Therapy-Related Myeloid Leukemia. Semin Oncol (2008) 35(4):418–29. doi: 10.1053/j.seminoncol.2008.04.012 PMC2600445 18692692

[96] Pedersen-Bjergaard J Andersen MK Christiansen DH. Therapy-Related Acute Myeloid Leukemia and Myelodysplasia After High-Dose Chemotherapy and Autologous Stem Cell Transplantation. Blood (2000) 95(11):3273–9. doi: 10.1182/blood.V95.11.3273.011k15_3273_3279 10828005

[97] Granfeldt Østgård LS Medeiros BC Sengeløv H Nørgaard M Andersen MK Dufva IH. Epidemiology and Clinical Significance of Secondary and Therapy-Related Acute Myeloid Leukemia: A National Population-Based Cohort Study. J Clin Oncol (2015) 33(31):3641–9. doi: 10.1200/JCO.2014.60.0890 26304885

[98] Metheny L Callander NS Hall AC Zhang MJ Bo-Subait K Wang HL. Allogeneic Transplantation to Treat Therapy Related MDS and AML in Adults. Transplant Cell Ther (2021) (11):923.e1-923.e12. doi: 10.1016/j.jtct.2021.08.010 PMC9064046 34428556

[99] Natelson EA Pyatt D. Acquired Myelodysplasia or Myelodysplastic Syndrome: Clearing the Fog. Adv Hematol (2013) 2013:309637. doi: 10.1155/2013/309637 24194760 PMC3806348

[100] Litzow MR Tarima S Pérez WS Bolwell BJ Cairo MS Camitta BM. Allogeneic Transplantation for Therapy-Related Myelodysplastic Syndrome and Acute Myeloid Leukemia. Blood (2010) 115(9):1850–7. doi: 10.1182/blood-2009-10-249128 PMC2832815 20032503

[101] Metafuni E Chiusolo P Laurenti L Sorà F Giammarco S Bacigalupo A. Allogeneic Hematopoietic Stem Cell Transplantation In Therapy-Related Myeloid Neoplasms (T-MN) of the Adult: Monocentric Observational Study and Review of the Literature. Mediterr J Hematol Infect Dis (2018) 10(1):e2018005. doi: 10.4084/mjhid.2018.005 29326802 PMC5760063

[102] Finke J Schmoor C Bertz H Marks R Wäsch R Zeiser R. Long-Term Follow-Up of Therapy-Related Myelodysplasia and AML Patients Treated With Allogeneic Hematopoietic Cell Transplantation. Bone Marrow Transplant (2016) 51(6):771–7. doi: 10.1038/bmt.2015.338 26752137

[103] Quintás-Cardama A Daver N Kim H Dinardo C Jabbour E Kadia T. A Prognostic Model of Therapy-Related Myelodysplastic Syndrome for Predicting Survival and Transformation to Acute Myeloid Leukemia. Clin Lymphoma Myeloma Leuk (2014) 14(5):401–10. doi: 10.1016/j.clml.2014.03.001 PMC4167474 24875590

[104] Zeidan AM Al Ali N Barnard J Padron E Lancet JE Sekeres MA. Comparison of Clinical Outcomes and Prognostic Utility of Risk Stratification Tools in Patients With Therapy-Related vs *De Novo* Myelodysplastic Syndromes: A Report on Behalf of the MDS Clinical Research Consortium. Leukemia (2017) 31(6):1391–7. doi: 10.1038/leu.2017.33 28111463

[105] Bono E McLornan D Travaglino E Gandhi S Gallì A Khan AA. Clinical, Histopathological and Molecular Characterization of Hypoplastic Myelodysplastic Syndrome. Leukemia (2019) 33(10):2495–505. doi: 10.1038/s41375-019-0457-1 30940907

[106] Karantanos T DeZern AE. Biology and Clinical Management of Hypoplastic MDS: MDS as a Bone Marrow Failure Syndrome. Best Pract Res Clin Haematol (2021) 34(2):101280. doi: 10.1016/j.beha.2021.101280 34404534

[107] Kahl C Leisenring W Deeg HJ Chauncey TR Flowers ME Martin PJ. Cyclophosphamide and Antithymocyte Globulin as a Conditioning Regimen for Allogeneic Marrow Transplantation in Patients With Aplastic Anaemia: A Long-Term Follow-Up. Br J Haematol (2005) 130(5):747–51. doi: 10.1111/j.1365-2141.2005.05667.x 16115132

[108] Konopacki J Porcher R Robin M Bieri S Cayuela JM Larghero J. Long-Term Follow Up After Allogeneic Stem Cell Transplantation in Patients With Severe Aplastic Anemia After Cyclophosphamide Plus Antithymocyte Globulin Conditioning. Haematologica (2012) 97(5):710–6. doi: 10.3324/haematol.2011.050096 PMC3342973 22180425

[109] Kennedy AL Shimamura A. Genetic Predisposition to MDS: Clinical Features and Clonal Evolution. Blood (2019) 133(10):1071–85. doi: 10.1182/blood-2018-10-844662 PMC6405331 30670445

